# Channel-Forming Activities in the Glycosomal Fraction from the Bloodstream Form of *Trypanosoma brucei*

**DOI:** 10.1371/journal.pone.0034530

**Published:** 2012-04-10

**Authors:** Melisa Gualdron-López, Miia H. Vapola, Ilkka J. Miinalainen, J. Kalervo Hiltunen, Paul A. M. Michels, Vasily D. Antonenkov

**Affiliations:** 1 Research Unit for Tropical Diseases, de Duve Institute, Université catholique de Louvain, Brussels, Belgium; 2 Department of Biochemistry, Biocenter Oulu, University of Oulu, Oulu, Finland; 3 Department of Pathology, University of Oulu, Oulu, Finland; Stanford University, United States of America

## Abstract

**Background:**

Glycosomes are a specialized form of peroxisomes (microbodies) present in unicellular eukaryotes that belong to the Kinetoplastea order, such as *Trypanosoma* and *Leishmania* species, parasitic protists causing severe diseases of livestock and humans in subtropical and tropical countries. The organelles harbour most enzymes of the glycolytic pathway that is responsible for substrate-level ATP production in the cell. Glycolysis is essential for bloodstream-form *Trypanosoma brucei* and enzymes comprising this pathway have been validated as drug targets. Glycosomes are surrounded by a single membrane. How glycolytic metabolites are transported across the glycosomal membrane is unclear.

**Methods/Principal Findings:**

We hypothesized that glycosomal membrane, similarly to membranes of yeast and mammalian peroxisomes, contains channel-forming proteins involved in the selective transfer of metabolites. To verify this prediction, we isolated a glycosomal fraction from bloodstream-form *T.brucei* and reconstituted solubilized membrane proteins into planar lipid bilayers. The electrophysiological characteristics of the channels were studied using multiple channel recording and single channel analysis. Three main channel-forming activities were detected with current amplitudes 70–80 pA, 20–25 pA, and 8–11 pA, respectively (holding potential +10 mV and 3.0 M KCl as an electrolyte). All channels were in fully open state in a range of voltages ±150 mV and showed no sub-conductance transitions. The channel with current amplitude 20–25 pA is anion-selective (*P*_K+_/*P*_Cl−_∼0.31), while the other two types of channels are slightly selective for cations (*P*_K+_/*P*_Cl−_ ratios ∼1.15 and ∼1.27 for the high- and low-conductance channels, respectively). The anion-selective channel showed an intrinsic current rectification that may suggest a functional asymmetry of the channel's pore.

**Conclusions/Significance:**

These results indicate that the membrane of glycosomes apparently contains several types of pore-forming channels connecting the glycosomal lumen and the cytosol.

## Introduction

*Trypanosoma brucei* is a parasite that belongs to the Trypanosomatidae family of the Kinetoplastea order of protists. The biology of *T. brucei* is under intensive investigation because of the medical and economical importance of these parasites as the causative agents of African trypanosomiasis, also known as sleeping sickness in humans and Nagana disease in cattle [1]–[3]. The complex life cycle of *T. brucei* involves its alternation between the insect vector (tsetse fly), where the replicative stage of the parasite is called procyclic form, and the blood of the mammalian host where the parasites differentiate into the so-called long-slender bloodstream form. The parasite's life cycle requires drastic metabolic changes in order to adapt to the environments encountered in the respective hosts [1]. It has been demonstrated that the glycolytic pathway is essential for *T. brucei*'s bloodstream form, since glycolysis is its only source of ATP synthesis. Remarkably, the first seven enzymes of glycolysis in the *Trypanosoma* species are localized in specific cellular organelles, glycosomes, where these enzymes may represent up to 90% of the total protein content [1], [4]–[6]. This is in contrast to cells of higher eukaryotes where all glycolytic enzymes are found in the cytosol. Glycosomes are members of the microbody family of organelles that also includes peroxisomes from mammals, plant leaves and yeasts as well as glyoxysomes from oil seeds [1], [6], [7]. All microbodies share common morphology and biogenesis, as well as some other properties, such as the absence of DNA and involvement in the metabolism of certain lipids [7]. However, the overall enzyme composition of the particles is different and in many cases varies depending on the nutritional source.

Usually in cells, the enzymes catalyzing the two steps in which ATP is invested at the beginning of the glycolytic pathway, hexokinase and phosphofructokinase, are allosterically regulated by their reaction products or other effectors. This regulation limits the so-called ‘turbo effect’, *i.e.* the uncontrolled activation of glycolysis by the net ATP that is produced at later steps of glycolysis. In contrast, an allosteric regulation of the activity of glycolytic ATP-consuming enzymes in Trypanosomatidae has not been detected [5], [6], [8], [9]. Instead, as has been shown recently [10], compartmentalization of glycolytic enzymes within the glycosomes of Trypanosomatidae prevents from the detrimental ‘turbo effect’ of an uncontrolled consumption of ATP at the initial steps of glycolysis. This is apparently achieved by formation of the two pools of ATP – glycosomal and cytosolic. The glycosomal pool of ATP needs to be strictly balanced by action of glycolytic enzymes consuming and producing ATP in glycosomes. The net production of ATP in glycolysis is catalyzed by the last enzyme of the pathway, pyruvate kinase, which is located in the cytosol [1], [2], [6], [10]. Separation of the first and second part of the glycolytic pathway between the two compartments thus predicts an important role for the glycosomal membrane in preventing free diffusion of ATP between the cytosol and the glycosomal lumen. How the glycosomal membrane is involved in the transfer of different metabolites, including ATP and other solutes such as glycolytic intermediates, is an unresolved issue.

As has been shown recently, some representatives of the microbody family, such as peroxisomes from plants, mammals, and yeasts, contain proteins that are able to form a general diffusion pore in the membrane [11]–[15]. In addition, experiments *in vitro* have revealed that the mammalian peroxisomal membrane is open to small solutes such as inorganic ions and most hydrophilic cellular metabolites, but selectively prevents diffusion of ‘bulky’ solutes, including cofactors (NAD/H, NADP/H and CoA) and ATP [16], [17]. One of the mammalian peroxisomal membrane channels, formed by the protein Pxmp2 from rodents, has been characterized at the molecular level [18]. The diameter of the channel's pore was estimated at 1.4 nm. This size is lower than the dimensions of ‘bulky’ metabolites but far above the diameter of small solutes. Therefore, the sieve properties of the peroxisomal membrane may be well determined by the pore size of the channel molecules. Considering the evolutionary relationship of peroxisomes and glycosomes one can predict the presence of channel-forming proteins also in the glycosomal membrane. These channels may allow an easy movement across the membrane of small solutes, including all intermediate metabolites of glycolysis, but prevent transfer of ATP and cofactors and thus promoting the maintenance of separate pools of these compounds in the cytosol and the glycosomal lumen.

Here we report results of an investigation of the channel-forming activities in purified glycosomal preparations isolated from bloodstream-form *T. brucei*. Three distinct activities were detected and their electrophysiological characteristics were analyzed using the planar lipid bilayer technique.

## Results

### Characterization of the isolated glycosomal fraction

**Figure 1:**
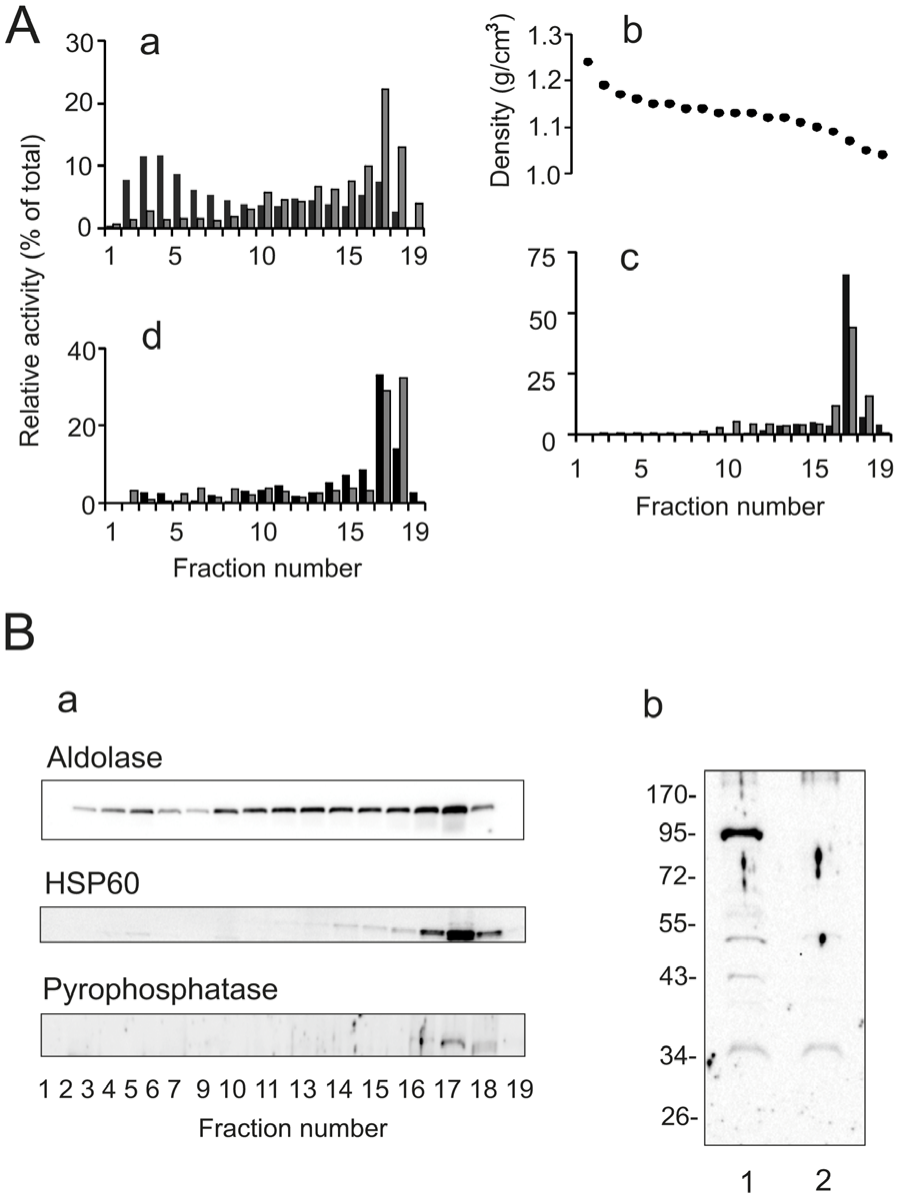
Isolation of glycosomes. A large granular fraction enriched in glycosomes and mitochondria as obtained by differential centrifugation was subjected to Optiprep density gradient centrifugation and the contents of the fractions obtained were analyzed by (**A**) marker enzyme activity measurements and (**B**) immunoblot analysis of marker proteins. (**A**) Activities of hexokinase (**a**, filled bars), FAD-dependent glycerol-3-phosphate dehydrogenase (**c**, filled bars), acid phosphatase (**c**, gray bars), mannosidase (**d**, gray bars), and α-glucosidase (**d**, filled bars) were measured. Protein content (**a**, gray bars) and density of the gradient (panel **b**) were also determined. The results obtained are expressed as the activity in each fraction relative to the total activity in the whole gradient. Enzyme (protein) recoveries varied between 78–112%. (**B**) Panel **a**: Proteins from equal volumes (70 µl) of each fraction (fractions 1–7, 9–11, and 13–19, see Figure 1**A**) were separated by SDS/PAGE and analyzed by western blotting using antibodies against markers for different organelles [aldolase as a glycosomal marker and heat shock protein 60 (HSP60) as a mitochondrial marker]. Panel **b**: Immunodetection of the acidocalcisome marker pyrophosphatase in the post-nuclear homogenate (line 1) and in the mitochondrial fraction (line 2). Molecular mass markers are indicated.

**Figure 2:**
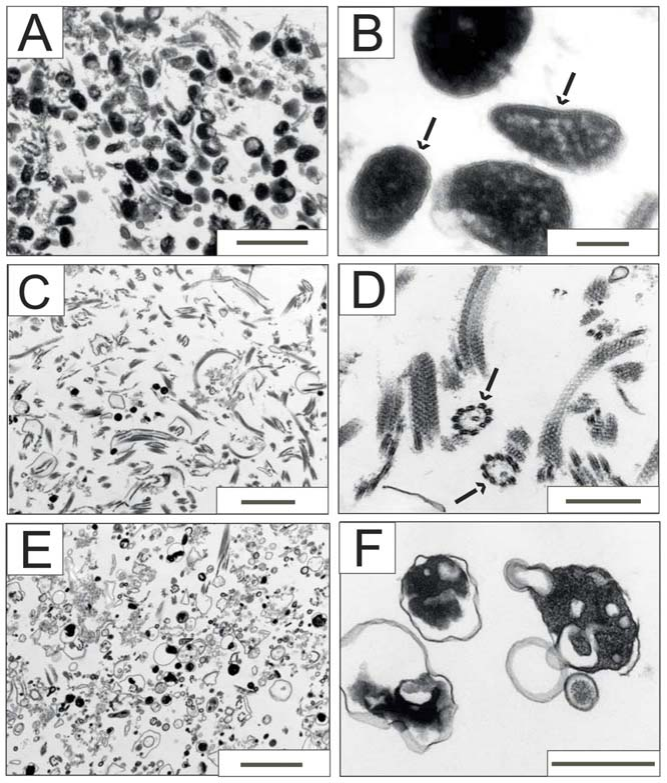
Electron microscopy of cellular organelles separated by Optiprep gradient centrifugation. Fractions enriched in glycosomes (fractions 2–5, see Figure 1A), fragments of flagella (fractions 8–11) or mitochondria and other organelles (fractions 15–18) were combined and processed for EM examination (see the Materials and methods section). (**A** and **B**) Isolated glycosomes shown at lower (**A**) and higher (**B**) magnifications. The fraction consists mostly of glycosomes. Some contamination by fragments of flagella is also visible. Importantly, fragments of flagella (paraflagellar rods and axonemes) show no sign of attachment to the flagellar membrane. Note the presence of intact glycosomes as electron-dense vesicles surrounded by a single membrane (marked by arrows in panel **B**). (**C** and **D**) Fractions enriched in flagella at low (**C**) and high (**D**) magnifications. One can see many paraflagellar rods in longitudinal section (**C**) and recognize flagellar axonemes (marked by arrows in panel **D**). Some glycosomes are also visible in panel **C**. (**E** and **F**) Composition of the fraction from the top of the Optiprep gradient that is enriched with mitochondria. Several types of organelles – mitochondria, lysosomes, lipid droplets, clathrin-coated vesicles, and components from the flagellar apparatus – can be observed. Note the shrinking of the mitochondrial inner membrane (see panel **F**) apparently due to osmotic misbalance. Scale bars: 2 µm (**C** and **E**); 1 µm (**A**); 0.5 µm (**D** and **F**), and 0.1 µm (**B**).

Glycosomes were purified from bloodstream-form cells of *T. brucei* using a two-step isolation procedure (Figure 1). Differential centrifugation was applied to obtain a large granular fraction enriched in glycosomes and mitochondria. This fraction was then subjected to Optiprep density gradient centrifugation to separate glycosomes from other cellular organelles. As has been shown previously, Optiprep is a highly effective medium for isolation of peroxisomes from different sources [17], [19] and glycosomes from *T. brucei* [20]. To preserve intactness of glycosomes we used poly(ethylene)glycol PEG 1500 (PEG 1500) as an osmoprotector expecting that, like in the case of mammalian [17] and yeast [13] peroxisomes, this compound may effectively prevent damage of the particles. Indeed, our preliminary experiments revealed that addition of PEG 1500 to the isolation medium significantly reduced the leakage rate of the glycosomal matrix enzyme hexokinase from the organelles (data not shown). Localization of glycosomes in the gradient fractions was monitored just after centrifugation by measuring hexokinase activity. According to the results obtained, the particles are detected near the bottom of the gradient and are also localized in the middle gradient fractions (Figure 1A, panel a). This broad distribution of glycosomes may reflect an appearance of the glycosomal ‘ghosts’ which, similar to the peroxisomal ‘ghosts’ [17], are formed due to the partial leakage of matrix proteins from damaged particles. The expected density of the glycosomal ‘ghost’ is lower than that of the intact organelle [17]. Purity of the glycosomal preparations was routinely estimated by analysis of the activity of marker enzymes and by immunodetection of marker proteins for different organelles (Figure 1). A significant portion of glycosomes (fractions 2–4 of the gradient, see Figure 1A) was well separated from the other cellular organelles although the yield was relatively low. This was an expected result since our isolation protocol was aimed to achieve the maximal purity of glycosomes that inevitably resulted in the lower yield of the particles. Enrichment of the glycosomal preparations as determined by hexokinase assays was about 10-fold relative to the specific enzyme activity in the granular fraction loaded on the gradient. In the glycosomal preparations we found only traces of the activities of markers for mitochondria (FAD-dependent glycerol-3-phosphate dehydrogenase) and flagellar membranes (acid phosphatase), indicating a low contamination of glycosomes by membrane fragments of these organelles which may be considered as the main potential sources of the contaminating channel-forming activities (Figure 1A, panel c). According to the distribution of marker enzymes activity, the glycosomal preparations contain less that 2% of the total amount of lysosomes (mannosidase) and endoplasmic reticulum (α-glucosidase) loaded on the gradient (Figure 1A, panel d). Considering the latter data, it is important to emphasize that the fraction loaded on the Optiprep gradient was obtained as a result of differential centrifugation of the postnuclear homogenate (see ‘Methods’ section). It is known that this procedure leads to partial purification of glycosomes especially to separation of these particles from endoplasmic reticulum and to a less extent from lysosomes [4], [21]. We also analyzed the purity of the glycosomal fraction by immunodetection of marker proteins for different organelles (Figure 1B, panel a). As expected, the glycosomal marker (aldolase) was detected in the bottom gradient fractions and in the other fractions containing glycosomes, while markers for mitochondria (heat shock protein 60, HSP60) and acidocalcisomes (vacuolar pyrophosphatase) were found exclusively in the top gradient fractions. The pyrophosphatase immunosignal was very weak even after long blot exposure (fractions 16–18, see Figure 1B, panel a). This is a predictable result since the bulk of acidocalcisomes was not sedimented with the large granular fraction during differential centrifugation. Indeed, immunodetection of pyrophosphatase at the same conditions of blotting revealed a strong signal at the corresponding protein size only in the postnuclear homogenate and no signal was found in the mitochondria-enriched fraction (compare lines 1 and 2 in Figure 1B, panel b). The purity of isolated glycosomes was also verified by electron microscopy (EM) (Figure 2). Consistent with the biochemical data, the EM images demonstrate that the preparations of glycosomes are highly enriched with these particles (Figure 2A). Most glycosomes are well preserved; they are filled with an electron-dense matrix and surrounded by a single membrane (Figure 2B). In the same preparations some fragments of the flagellar apparatus (paraflagellar rods and axonemes) can be detected. As shown by a careful analysis of the images, these structures are not connected to the flagellar membranes, which corroborate the data obtained in the enzyme activity assays performed for the flagellar membrane marker, acid phosphatase (see Figure 1A, panel c). One may therefore expect that the low contamination of the glycosomal fraction by protein fragments of the flagellar apparatus should not affect the results of the detection of channel-forming activities since these activities belong to proteins which are localized in the membrane structures. We also conducted EM of fractions in the middle (fractions 8–11, see Figure 1) and the top (fractions 15–18) of the Optiprep gradient. The middle fractions showed a high enrichment of fragments of the flagellar apparatus (Figure 2C and 2D), whereas the top fractions contained a complex mixture of different membrane-bounded organelles with mitochondrial vesicles as a predominant constituent (Figure 2E and 2F).

### Multiple channel recording (MCR) of the isolated organelles

**Figure 3:**
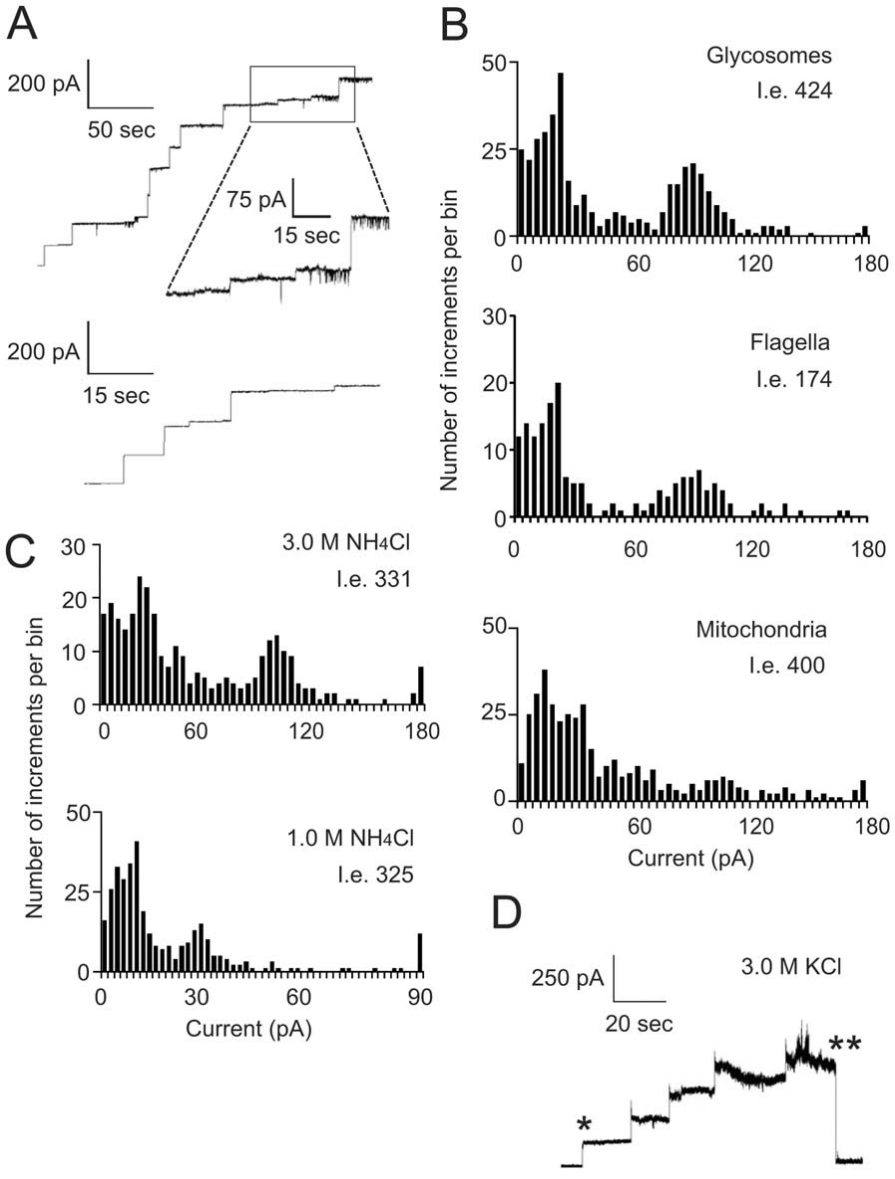
Detection of channel-forming activities in subcellular fractions. Fractions 2–4 (glycosomes), 8–11 (fragments of flagella), and 15–18 (mitochondria) from Optiprep density gradients (see Figure 1A) were combined and treated with Genapol X-080 to solubilize membrane proteins (see the Materials and methods section). After sedimentation of insoluble material, aliquots of the resulting supernatants were used for MCR (**A**–**C**) or SCA (**D**). (**A**) Traces of the current monitoring in the presence of glycosomal (upper panel) or mitochondrial (lower panel) preparations. The middle trace represents a timescale-expanded current recording of the upper trace. The bath solution contained 3 M KCl and the applied voltage was +10 mV. (**B**) Histograms of insertion events registered in subcellular fractions (see panel **A**). Bin size is 4.0 pA. The total number of insertion events (I.e.) is indicated. Here and in Figure 3 C (upper panel) all insertion events with current increments over 180 pA (for Figure 3C, lower panel −90 pA) are combined in one bin (180 pA or 90 pA, respectively). Note that the amount of insertion events in the flagella fraction (see **B**, middle panel) is lower than that observed in other fractions. This is mainly due to low channel-forming activity (per protein content) in the preparations of this fraction. For the sake of compatibility we used the same amounts of protein for measurements in different fractions. (**C**) Histograms of insertion events detected for glycosomal preparations using NH_4_Cl as the electrolyte. Bin size: 4 pA (upper panel) or 2 pA (lower panel). See legend to Figures 3A and 3B for other details. (**D**) Trace of the current monitoring using the glycosomal fraction (initial holding potential +10 mV) indicating the insertion (marked by one asterisk) of a large-conductance channel that spontaneously closed (marked by two asterisks) after stepwise (each step is +10 mV) increase in the holding potential up to 50 mV.

MCR is a useful analytical tool to describe an overall pattern of channel-forming activities in certain membrane preparations. This method is especially suitable for samples containing several types of pore-forming proteins. It also helps to establish optimal experimental conditions for revealing activity of the channels. We initially attempted to conduct MCR using 1.0 M KCl as a bath solution. While the activity in the glycosomal fraction was evident, the amount of insertion events was relatively low and the inserted channels were mainly unstable, frequently showing an intensive flickering (data not shown). Therefore, we increased the ionic strength of the bath solution up to 3.0 M KCl. This led to the appearance of more stable channel-forming activities (Figure 3A) that allowed quantitative analysis of the whole set of insertion events using histograms which indicate insertion frequency relative to current amplitude at a certain holding potential (Figure 3B). This approach was applied for comparative analysis of pore-forming activities in the preparations of glycosomes (Figure 3B, upper panel), flagella (Figure 3B, middle panel) and mitochondria (Figure 3B, lower panel). The conductance pattern registered in the glycosomal preparations was clearly different from that of the mitochondrial fraction containing in addition to mitochondria some other membrane-bounded organelles. This may indicate that glycosomes contain their own pool of channel-forming proteins and the registered activities are not caused by contaminating proteins from other organelles. Comparative MCR of glycosomal and flagellar preparations revealed similar patterns of current amplitudes, whereas the frequency of insertion events with the flagellar fraction was much lower. Remarkably, we did not register any noticeable number of current increments with amplitudes characteristic for only flagellar preparations. These observations are in line with the prediction that the channel-forming activity in the flagellar fractions is due to their contamination with glycosomes.

Next, we studied conditions favourable for detection of the channel-forming activities and tried to register these activities using different bath solutions (NaCl, LiCl, NH_4_Cl, potassium acetate, sodium glycolate, and sodium phosphate, pH 7.4). The best results were obtained using NH_4_Cl as the electrolyte (Figure 3C). A high frequency of insertion events was observed not only at 3.0 M NH_4_Cl (Figure 3C, upper panel), but also at 1.0 M NH_4_Cl (Figure 3C, lower panel). The overall patterns of insertion events registered at +10 mV using 3.0 M KCl and 3.0 M NH_4_Cl as bath solutions were similar (compare Figure 3B, upper panel and 3C, upper panel). They showed two predominant types of channel-forming activities with average current amplitudes of 20–25 pA and 70–80 pA, respectively. As expected, lowering of the ionic strength of the electrolyte from 3.0 M to 1.0 M NH_4_Cl was accompanied by a corresponding decrease in the registered conductance levels (compare Figure 3C, upper panel and Figure 3C, lower panel). This is a characteristic feature of non-selective channels forming water-filled pores in the membrane [22], [23].

To proof a membrane localization of the channels, the glycosomal preparations were treated by sonication and the membrane fragments were separated from matrix proteins by sucrose density gradient centrifugation. As expected, the channel-forming activity was only detected in the membrane preparations (data not shown). To analyze a potential role for protein SH-groups in modulation of the channels activity, glycosomal preparations were pre-incubated with 5 mM dithiothreitol (DTT) or the reducing agent was added into the bath solutions (2 mM DTT, final concentration). We did not detect any effect of DTT on the conductivity pattern of analyzed channels (data not shown). Nevertheless, we performed standard activity measurements in the presence of 2.0 mM DTT to preserve any SH-groups from oxidation during prolonged incubation periods. A moderate increase in the applied potentials (up to 60 mV) did not influence the conductivity pattern of glycosomal preparations, but strongly reduced the amount of high-conductance insertion events registered in the mitochondrial fraction (data not shown). More detailed investigation of this phenomenon using single channel analysis (SCA) showed that, contrary to high-conductance activity in glycosomal preparations (see below), the mitochondrial channel is spontaneously gated at holding potentials above 40–50 mV (Figure 3D). This difference in properties of high-conductance channels was routinely used in the following experiments to distinguish them from each other.

### Single channel analysis (SCA) of a high-conductance channel

**Figure 4:**
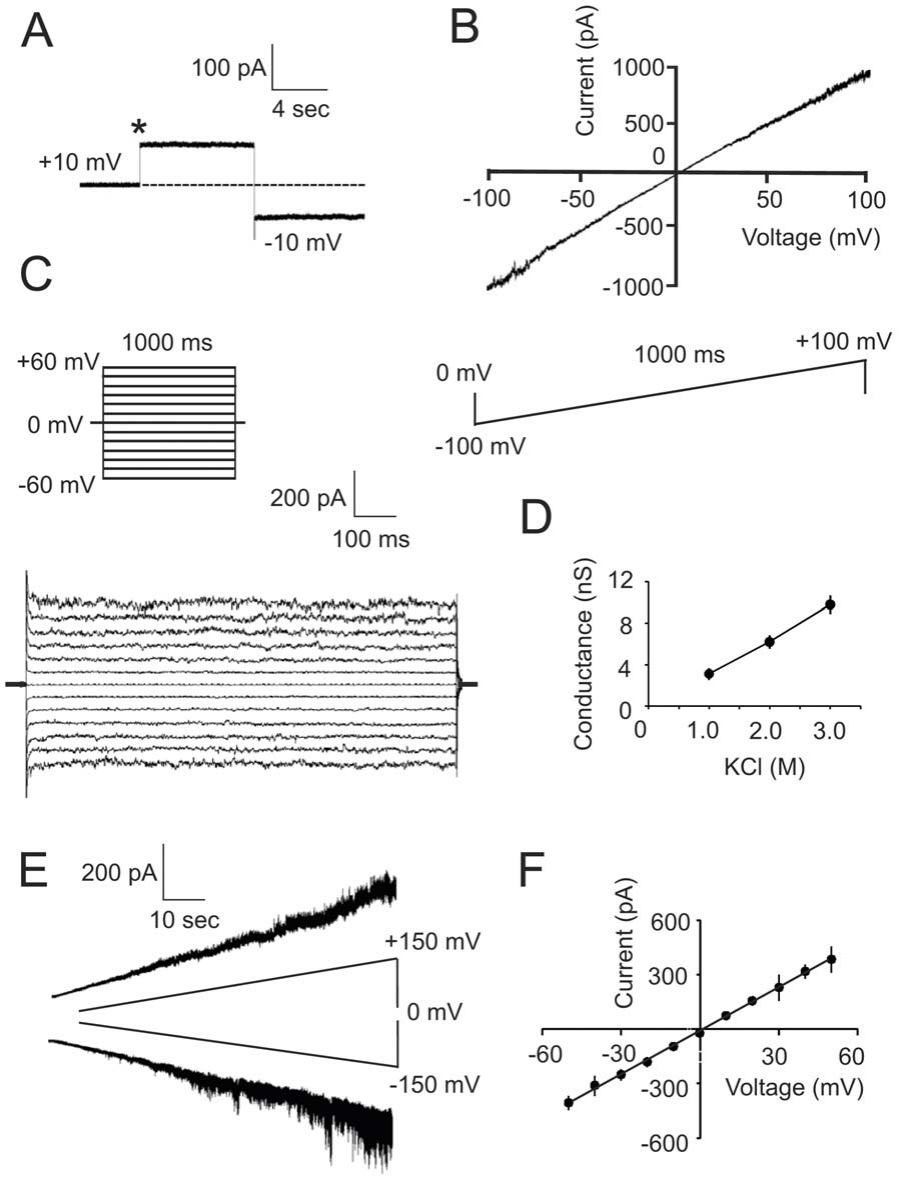
SCA of a high-conductance channel. (**A**) Current trace of a single high-conductance channel. The insertion event (marked by an asterisk) was registered at +10 mV and the applied voltage was then switched to −10 mV. The dashed line indicates the current level (zero) before insertion of the channel. The data in panels **A**, **B**, and **C** were collected using 3 M KCl as the electrolyte. (**B**) Current trace of the channel in response to the indicated voltage-ramp protocol. Note the near linear dependence of the current on the applied voltage. (**C**) Single channel currents in response to the indicated voltage-step protocol. (**D**) Dependence of the single channel conductance on the KCl concentration. After detection of a single channel insertion using 3 M KCl as bath solution (holding potential +10 mV), the electrolyte was diluted and registration of the current amplitudes of the same channel was conducted at 2.0 M and 1.0 M KCl, respectively. Data points are mean±SD for at least 4 independent measurements. (**E**) Current traces of a single channel in response to a low-speed linear increase (upper trace) or decrease (lower trace) of the holding potential. The bath solution contained 1.0 M NH_4_Cl, 20 mM Tris-Cl, pH 7.8, and 2 mM DTT at both sides of the membrane. Note that the channel was still open even at hyperpolarizing holding potentials of ±150 mV. (**F**) Current-voltage relationship of the high-conductance channel under asymmetric salt conditions: 3.0 M KCl *trans*/1.5 M KCl *cis* compartment. The insertion of a single channel was detected at 3 M KCl at both sides of the membrane and at a voltage of +10 mV, then the electrolyte concentration in the *cis* compartment was decreased by dilution and an initial current recording was conducted at zero potential followed by stepwise (±10 mV) change of the applied voltage. Data points are mean±SD, n = 4–5. Bars in some cases are smaller than symbols.

We applied SCA for detailed electrophysiological characterization of the channel-forming activities registered using MCR (see above). The most prominent of these activities was a high-conductance channel (Figure 4A) which comprises more than 25% of the total number of insertion events registered by MCR (see Figure 3B, upper panel). The channel inserted in the membrane was usually stable in the fully open conformation during the entire period of registration (minutes) at different holding potentials. This was confirmed using voltage-ramp (Figure 4B) and voltage-step (Figure 4C) protocols. The results indicate a near linear dependence of the channel current on the voltage with a slope conductance of Λ = 9.2±0.6 nS, n = 8 (3.0 M KCl on both sides of an artificial membrane). The conductance of a single channel was linearly decreased following dilution of the bath solution from 3.0 M KCl to 2.0 M then1.0 M KCl (Figure 3D). The high conductance of the channel and linear dependence on the KCl concentration suggest an electrochemical current flow through a porin-like structure forming a hole in the membrane which is filled with water [22], [23]. The channel was resistant to gating even at extreme holding potentials (Figure 4E), distinguishing it from the voltage-dependent anion channel of the outer mitochondrial membrane [24]. The probability of the channel to be open was *P*_open_≈0.9 between *V*_hold_ = +150 mV and *V*_hold_ = −150 mV. Only a single conductance state was detected. The reversal potential of the channel in asymmetric KCl solutions (3.0 M KCl *trans*/1.5 M KCl *cis*) was *E*_rev_ = +1.14 mV indicating that this channel has only limited preference for cations over anions, *P*_K+_/*P*_Cl−_ratio ∼1.15.

### Low-conductance channel showing current rectification

**Figure 5:**
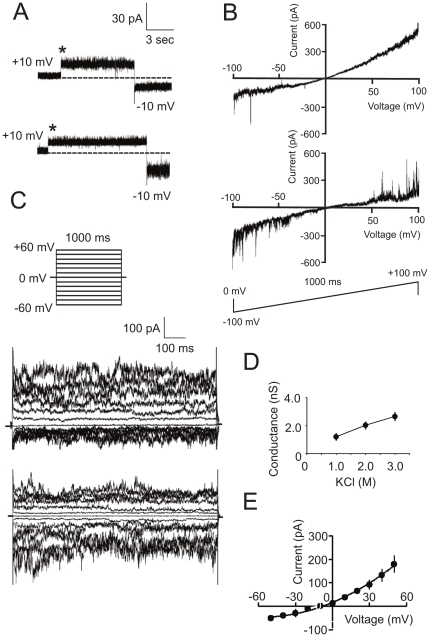
SCA of a low-conductance channel. (**A**) Current traces of two low-conductance channels. The bath solution (**A–C**) contained 3 M KCl at both sides of the membrane. See legend to Figure 4**A** for other details. Note that two types of channels were registered. Most of them showed a larger current amplitude at +10 mV than at −10 mV (upper trace). In contrast, some channels displayed an opposite trend (lower trace). (**B** and **C**) Current traces of the channels in response to the indicated voltage-ramp (**B**) and voltage-step (**C**) protocols. Most detected channels displayed a current rectification at negative holding potentials (upper panels). However, in a few cases the rectification was observed at positive holding potentials (lower panels). (**D**) Dependence of the low-conductance channel activity on the electrolyte concentration. The channel insertion was registered at a holding potential of +10 mV using 3 M KCl as a bath solution. After confirming that the channel shows current rectification at negative voltages by application of a voltage-ramp protocol, the electrolyte in the chambers was diluted to 2.0 M or 1.0 M KCl and the current amplitudes were measured at +10 mV. Data are mean±SD, n = 4–5. (**E**) Ion selectivity of the low-conductance channel. See legend to Figure 4**F** for details. The current-voltage relationship of channels (rectification at negative voltages) was validated using a voltage-ramp protocol.

In addition to high-conductance channel-forming activity, the MCR experiments revealed an abundant low-conductance activity in glycosomal preparations (see Figure 3, upper panel). Similarly, the insertion events with current amplitudes 20–25 pA at a holding potential +10 mV and an electrolyte concentration of 3.0 M KCl were frequently detected by SCA. Surprisingly, a switch of the applied voltage from +10 mV to −10 mV was accompanied by a significant decrease in the current flow through a single channel (Figure 5, upper panel). However, in a few cases the same change in the holding potential led to the opposite result – an increase in the current amplitude (Figure 5A, lower panel). These results may indicate that the low-conductance channels show current rectification. Indeed, analysis of the current-voltage relationships using voltage-ramp (Figure 5B) and voltage-step (Figure 5C) protocols confirmed this prediction. Within the whole set of the activities registered by SCA, 48 out of 56 low-conductance channels displayed rectification at negative voltages (Figure 5B and 5C, upper panels). The mean chord conductance of this channel, as deduced from the results in Figure S1A, was 2.8±0.4 nS at +50 mV and 1.4±0.3 nS at −50 mV. Most channels showed an intensive flickering at holding potentials over +40 mV. However, we did not detect any sub-conductance states of the channel that preserved an open conformation at a range of voltages ±150 mV (Figure S1B). Interestingly, at holding potentials below −100 mV the channels partially lost their rectification ability (see Figure S1, panel B2). The dependence of current amplitudes on the strength of an electrolyte solution deviated moderately from a linear curve towards lower conductance rates especially at high KCl concentrations (Figure 5D). At asymmetric electrolyte conditions (3.0 M KCl *trans*/1.5 M KCl *cis*) the reversal potential of the channel was *E*_rev_ = 9.0 mV, indicating anion selectivity with *P*_K+_/*P*_Cl−_ ratio 0.31 (Figure 5E). Rectification by the low-conductance channels may indicate a longitudinal asymmetry of the channel's pore especially regarding the distribution of charged amino acids [22], [23], [25]. The fact that most channels show rectification at negative voltages suggests that incorporation of the channels is a directional process. To verify this prediction we tried to introduce the channels not from the *trans* side (standard conditions), but from the opposite, *cis* side of an artificial membrane. This was accompanied by predominant insertion of channels showing rectification at positive voltages (Figure S1C).

### Electrophysiological properties of a very-low-conductance channel

**Figure 6:**
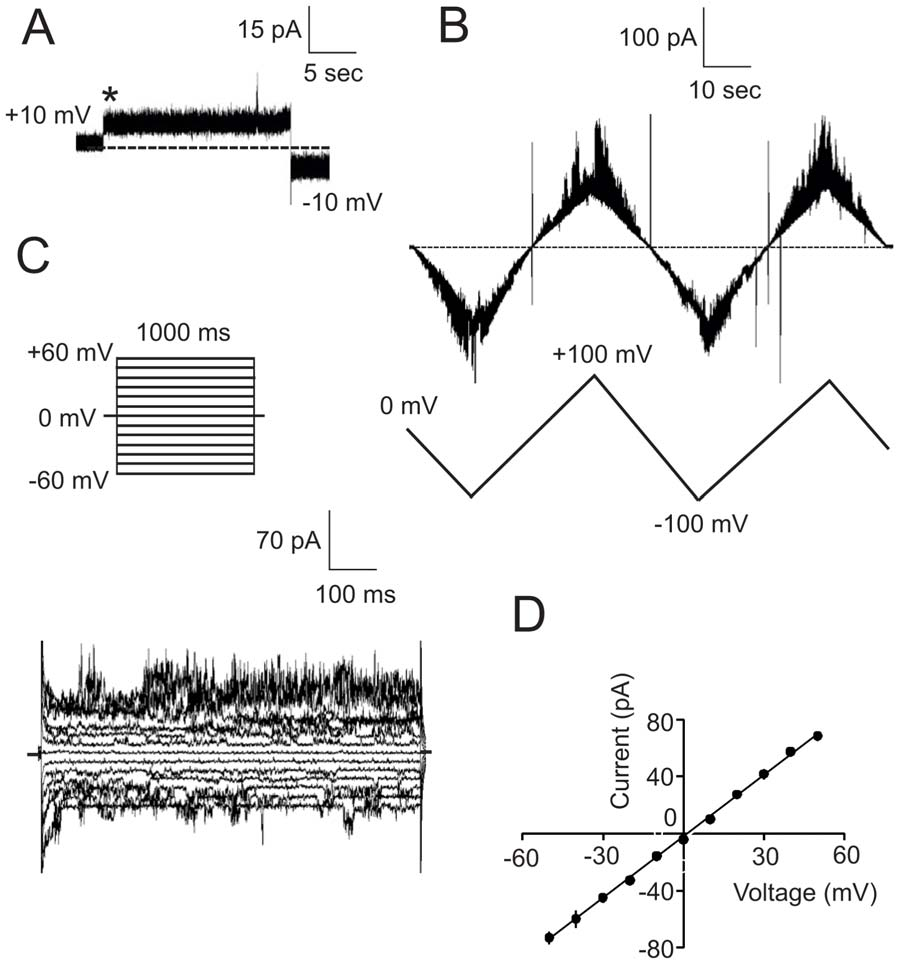
SCA of a very-low-conductance channel. (**A**) Current recording of a single very-low-conductance channel. The bath solution (panels **A**, **B**, and **C**) contained 3 M KCl. See legend to Figure 4**A** for other details. (**B**) Current trace of the channel in response to the shown voltage-ramp protocol. Dotted line indicates the current level at zero holding potential. Note the near linear dependence of the current on the applied voltage. (**C**) Current traces of a single channel in response to the indicated voltage-step protocol. (**D**) Ion-selectivity of the channel. See legend to Figure 4**D** for details.

The third abundant group of channel-forming activities in the glycosomal preparations besides the high- and low-conductance channels was represented by channels showing current amplitude of 8–11 pA (3.0 M KCl, +10 mV; see Figure 6A). This activity comprises more than 15% of the total number of insertion events registered by SCA in glycosomal preparations. The activity is near linearly dependent on the KCl concentration (data not shown). The current-voltage relationship measured at symmetric salt conditions (3 M KCl) revealed a slope conductance of Λ = 0.98±0.4 nS, n = 4 (data not shown). Contrary to the low-conductance activity (see above), this very-low-conductance channel showed no signs of current rectification. When applying both voltage-ramp (Figure 6B) and voltage-step (Figure 6C) protocols, the response of the channel's current to voltage modulations was close to linear. We did not detect gating of the channel and appearance of any sub-conductance states at low-speed linear change of the holding potential from zero to 150 mV in both, positive and negative, directions (data not shown). The reversal potential of the very-low-conductance channel in asymmetric salt concentrations (3.0 M KCl *trans*/1.5 M KCl *cis*) was *E*_rev_ = +2.0 mV that gives the *P*_K+_/*P*_Cl−_ ratio ∼1.27. Therefore, the channel is slightly cation-selective.

### Super-large-conductance channel-forming activities

During MCR of glycosomal preparations at standard conditions (3 M KCl or 3 M NH_4_Cl, +10 mV) channel-forming activities with current amplitudes over 180 pA were occasionally detected (Figures 3B and 3C). The activities mainly with conductance of ∼24.0, 32.0, and 50.0 nS (3 M KCl) were also registered by means of SCA (Figure S2). Some of these channels were stable in their open confirmation (Figure S2A), but most of the activities displayed an irregular flickering (Figure S2B), indicating fast transition between open and closed states. The average mean lifetime of the fully open, unstable channels was remarkably low (τ_open_<50 ms). Attempts to apply a voltage-ramp protocol to the stable super-large-conductance activities usually led to the appearance of multiple sub-conductance levels (Figure S2C), indicating that several channel-forming molecules may form clusters which are more or less resistant to treatment with detergents. Low abundance of the super-large-conductance channels precluded a detailed analysis of their properties.

## Discussion

Glycosomes of *T. brucei* are highly specialized subcellular organelles focused on the conversion of the main nutrient for the bloodstream form of the parasite, glucose, into 3-phosphoglycerate and under anaerobic conditions, also glycerol. The 3-phosphoglycerate is further metabolized in the cytosol to pyruvate, a metabolic end-product for this parasite, with concomitantly a net production of ATP [1]. However, the spectrum of metabolic activities of glycosomes is not limited to only glycolysis but also includes such diverse pathways as β-oxidation of fatty acids, ether-lipid and squalene biosynthesis, the pentose-phosphate shunt, purine salvage, synthesis of pyrimidines, energy homeostasis, and others, although many of these non-glycolytic activities are largely or entirely repressed in the bloodstream-stage of these trypanosomes [1], [26]. The diversity of metabolic pathways in glycosomes raises questions concerning the ability of the glycosomal membrane to cope with the transfer of different metabolic intermediates between glycosomal lumen and cytosol. Like other membranes impermeable to solutes, such as the inner mitochondrial or the plasma membranes, the membrane of glycosomes may contain many transporter proteins specific for certain metabolites. However, to our knowledge, only one of the 24 solute transporters of the mitochondrial carrier family of trypanosomes with still unidentified substrate specificity, MCF6, has been documented in the glycosomal membrane of bloodstream-form cells, whereas in procyclic cells it was located in the mitochondrial membrane [27], [28]. Information about some other solute transporters requires further clarification since the isolated glycosomal fraction where these transporters were detected by using a proteomic approach may have been contaminated by mitochondria [20]. In contrast, if the glycosomal membrane is open to solutes, it should, like the outer mitochondrial membrane or outer membrane of Gram-negative bacteria, contain pore-forming channels with low selectivity towards the chemical nature of the transported metabolites. Indeed, as shown in this report, such channel-forming activities were readily detected in solubilized membranes from preparations of glycosomes isolated from bloodstream-form *T. brucei*. It nevertheless should be emphasized that any researcher who use an isolated fraction of certain organelles faces a problem of contamination of this fraction by other subcellular particles. Therefore, we tried to exploit additional approaches to verify link between the detected channel-forming activities and glycosomes. In this context it is important that the conductivity pattern of the channels in glycosomal preparations determined by MCR was distinctly different from that obtained with membrane protein preparations from the mitochondrial fraction which harbours, among other channels, the mitochondrial outer membrane voltage-dependent anion channel (VDAC). Due to its abundance, this channel is usually considered as a major mitochondrial contaminant of different subcellular fractions. Interestingly, we detected in our mitochondrial preparations a high-conductance channel-forming activity with gating properties similar to VDAC (see Figure 3D). However, only traces of this activity were found in the glycosomal preparations. This observation coincides with the results of the purity analysis of the glycosomal fraction that showed very low contamination by membrane-bounded organelles other than glycosomes such as mitochondria, lysosomes, and acidocalcisomes as well as fragments of the endoplasmic reticulum and flagellar membranes (see Figure 1). As was revealed by EM examination, the purified preparations of glycosomes contain some fragments of the flagellar apparatus (paraflagellar rods and axonemes). However, these fragments are not connected to the flagellar membrane which might have been a potential source of the channel-forming activity contamination. As a whole, our experiments have revealed the channel-forming activities in purified glycosomal preparations. This can be considered as an indication on the presence of channel proteins in glycosomal membrane. However, further investigation is required to reveal the molecular nature of the channel-forming activities described here.

Three main channel-forming activities (high-, low-, and very-low-conductance) were detected in the glycosomal preparations. We did not find evidence for the possibility that any of these activities are in fact subconductance states of the other registered channels. In particular, the activities did not show spontaneous transitions between different conductance levels and were highly resistant to gating even at extreme holding potentials. This may lead to suggestion that not only one, but several distinct pore-forming proteins are apparently localized in the glycosomal membrane. The same conclusion has been made recently after analysis of the channel-forming activities in preparations of mammalian [12], [18] and yeast [13], [14] peroxisomes. Similar properties have been previously attributed to the outer membranes of Gram-negative bacteria and chloroplasts. Both these membranes contain different types of pore-forming proteins [29], [30]. The reason for localization of several distinct channels in the same membrane is not entirely clear. Some of them (so-called specific porins) show a preference for transfer certain compounds [29]. The function of others is under regulatory control by corresponding metabolites [30]. The latter property is especially evident for channels displaying an intrinsic rectification. Therefore it is highly probable that the intrinsically rectifying low-conductance channel detected in our experiments (see Figures 5 and S1) is formed by an allosterically regulated protein.

Our observation of the super-large-conductance channel-forming activities in glycosomal preparations is intriguing since it may be linked to the formation of a large and highly dynamic pore by the peroxisomal importomer which is involved in the transmembrane transfer of the newly synthesized, completely folded proteins into the matrix of peroxisomes [31]. The high conductance and the rare appearance of the super-large conductance channels may indicate that they are indeed the peroxisomal importomers. On the other hands, these activities may reflect formation - natural or artificial - of the channel clusters that is a common feature of hydrophobic, membrane-bound proteins.

The most intriguing question arising from our study is if and how the apparent glycosomal channels may be involved in regulation of glycolysis. The relatively high conductance and the long-lasting open states of the channels predict that they form pores in the membrane filled with water, *i.e.* belong to family of so-called non-selective channels [22], [24], [29]. The sieve properties of each such channel are mainly determined by the dimension of its pore. Our previous results on mammalian [15] and yeast [13] peroxisomes revealed that the membrane of these particles is highly selective towards the size of transported molecules. The peroxisomal channels allow transmembrane diffusion of solutes with molecular mass up to 300–400 Da but are unable to transfer ‘bulky’ metabolites such as ATP and some cofactors (NAD/H, NADP/H, CoA) whose molecular mass exceeds 500 Da. The sieve properties of peroxisomal channels apparently determine the formation of two independent pools, peroxisomal and cytosolic, of ‘bulky’ metabolites. In contrast, the peroxisomal lumen and the surrounding cytosol may share a common pool of small solutes. If the same rules are applicable to glycosomes, one can suggest a physical separation of glycosomal and cytosolic pools of ATP by the glycosomal membrane that might be sufficient and important to prevent from ‘turbo effect’ of uncontrolled ATP production (see ‘Introduction’ for further details). The proposed model does not require a multitude of glycosomal transporters specific for each small solute that has to be translocated, since these solutes would be able to use the channels to overcome the membrane barrier. However, transporters specific for at least some ‘bulky’ solutes are necessary. We are tempted to speculate that the MCF6 protein [27], if indeed its location in the glycosomal membrane can be confirmed, is involved in the transfer of some ‘bulky’ solute(s).

The presence of pore-forming proteins with low selectivity towards small solutes in the glycosomal membrane seems, at first glance, inconsistent with the systems biology analysis that demonstrated moiety conservation relations within the glycosomes not only for adenine nucleotides (ATP+ADP+AMP) and nicotinamide adenine nucleotides (NAD^+^+NADH), but also for all the phosphorylated intermediates involved in the glycolytic reactions occurring within the organelles [32]. Moreover, pulse-labeling experiments with radioactive glucose indicated that the exchange of phosphorylated intermediates between the two cellular pools is 60 times slower than the labeling of pyruvate [33]. Furthermore, it has been experimentally shown that concentrations of intermediates can increase to very high levels within the trypanosome if glycosomal enzymes are relocated from glycosomes to the cytosol [10]. These are all indications of a low rate of release of these intermediates out of glycosomes. In view of the results presented in this paper, one may wonder if this process is not so much the result of an impermeability barrier created by the glycosomal membrane, but rather the consequence of so-called Donnan equilibrium that may exist between glycosomal lumen and surrounding cytosol. The mechanism of Donnan equilibrium in biological systems is well known [34] and apparently responsible for the formation of a pH gradient across the mammalian and yeast peroxisomal membranes [6], [18] and outer mitochondrial membrane [35]. The low release of the intermediates from a multienzyme complex within the glycosomes in which substrate channeling might occur can also be considered. Indeed, there are indications for a strong association of the enzymes found at high density within the organelles, but so far channeling has not been demonstrated. This possibility is currently under investigation.

Which protein molecules determine the glycosomal channel-forming activities remains to be established. One of the channel-forming proteins in mammalian peroxisomes, Pxmp2, has recently been described [18]. The isolated protein forms homotrimer which is active as a channel in *in vitro* experiments and show a conductance of 1.3 nS in 1.0 M KCl. The channel is weakly cation-selective and shows no voltage dependence. An estimated diameter of the channel's pore is ∼1.4 nm, intermediate between the dimensions of small and ‘bulky’ solutes. Surprisingly, deletion of Pxmp2 in mice by disruption of the corresponding gene did not lead to the development of a severe phenotype, indicating redundancy of the channel function in mammalian peroxisomes. Indeed, in the peroxisomal preparations purified from liver of *Pxmp2^−/−^* mice some of the channel-forming activities were still registered [18]. Therefore, channel proteins other than Pxmp2 may be expected in the peroxisomal membrane. Good candidates for this role may be members of the Pex11 family of proteins. The family consists of three proteins localized in the mammalian and yeast peroxisomal membranes [36], [37]. Remarkably, trypanosomal homologues of this protein family (PEX11, GIM5A, and GIM5B) are also main components of the glycosomal membrane [38] that apparently does not contain proteins detectably homologous to mammalian Pxmp2 since we failed to find any sequence in the *T. brucei* genome with significant similarity to the *Pxmp2* gene (Antonenkov, unpublished results). The Pex11 family members are usually considered as proteins involved in the biogenesis of peroxisomes [36]–[38]. However, knock-out of yeast *Pex11* is accompanied by evident disruption of the transport function of the peroxisomal membrane [39]. The channel-forming activity of yeast and mammalian Pex11 proteins has recently been observed and is currently under investigation (Grunau et al., manuscript in preparation).

In conclusion, we described here channel-forming activities in purified glycosomal preparations isolated from the bloodstream form of *T. brucei.* The channels which are most probably the glycosomal membrane constituents may be involved in the transmembrane transfer of metabolic intermediates and could play an important role in creating conditions that prevent deregulation of the glycolytic pathway and energy balance in the *T. brucei* cells.

## Materials and Methods

### Chemicals

Optiprep density gradient medium, a 60% w/v, solution of Iodixanol in water was from Axis-shield PoC AS. A cocktail of protease inhibitors was from Fermentas. An artificial lipid bilayer former, diphytanoyl phosphatidylcholine, was obtained from Avanti Polar Lipids Inc. Genapol X-080 was from Fluka and Fos-choline-10 from Affymetrix. Reagents for electron microscopy were from Electron Microscopy Sciences. All other chemicals were from Sigma.

### Growth and isolation of trypanosomes

Monomorphic long-slender bloodstream-form cells of *T. brucei* strain Lister 427 (cell line 449) were grown in 300 g Wistar rats. Blood was collected from animals showing high parasitaemia (between 10^8^ and 10^9^ trypanosomes/ml) by cardiac puncture under ether anesthesia. Trypanosomes were separated from blood constituents by ion-exchange chromatography on DEAE-cellulose [40] and washed twice by centrifugation at 1000 *g* for 10 min using phosphate-buffered saline (PBS) containing 50 mM D-glucose. A final wash step prior to glycosome purification was performed using homogenization buffer (250 mM sucrose, 1 mM EDTA, 0.1%, v/v, ethanol, 5 mM MOPS, pH 7.2 and 12%, w/v, PEG 1500). PEG 1500 was used to prevent osmotic damage of glycosomes [17]. All animal care and handling procedures were approved by the committee on animal experimentation of the Université catholique de Louvain (Belgium): 2006/UCL/MD/037 BCHM (TROP) “Etude de la biochimie, de la biologie cellulaire et de la biologie moléculaire des protozoaires responsables des maladies parasitaires chez l'homme et particulièrement des protozoaires de la famille *Trypanosoma* (rats and mice)”.

### Purification of glycosomes

Trypanosomes were harvested by centrifugation for 10 min at 1000 *g* and the cell pellet (approximately 5.0×10^10^ cells) was subjected to grinding with silicon carbide (325 mesh) [41] in the presence of homogenization buffer supplemented with a cocktail of protease inhibitors (total volume 2 ml). The disruption of parasites was followed by phase-contrast microscopy and continued till at least 95% of the cells were broken. After dilution of the resulting mixture with homogenization buffer (final volume 10 ml) the silicon carbide was removed by centrifugation at 100 *g* for 3 min. The homogenate obtained was centrifuged twice at 1500 *g* for 10 min to remove nuclei and cellular debris. The postnuclear supernatant was then centrifuged at 17 000 *g* for 15 min to sediment mitochondria and glycosomes. The pellet was resuspended in homogenization buffer using a Dounce homogenizer and 5 ml of the suspension were loaded on two preformed linear 20%–35%, v/v, Optiprep gradients (33 ml each) mounted on top of a 3.5 ml 50%, v/v, Optiprep cushion according to the manufacturer's instruction (Optiprep Application Sheet S09, Axis-shield). The gradients were prepared with homogenization buffer containing no sucrose and PEG 1500. The samples were centrifuged in a vertical VTi-50 (Beckman) rotor at 100 000 *g* for 120 min at the slow acceleration and deceleration mode. Fractions of 2 ml were collected from the bottom of each tube and were used immediately for analysis of the activities of marker enzymes or were frozen at −70°C. Disruption of glycosomes by sonication and separation of membrane fragments from matrix proteins by sucrose density gradient centrifugation were performed as described [17].

### Measurement of enzyme activities

Spectrophotometric measurements of the activity of marker enzymes for different cellular organelles were conducted according to standard procedures. Activity determination of hexokinase [42] and FAD-dependent glycerol-3-phosphate dehydrogenase [43] was used to localize in Optiprep gradient fractions the glycosomes and mitochondria, respectively. Mannosidase activity (lysosomal marker) was detected with 4-nitrophenyl-D-mannopyranoside as a substrate [44]. α-Glucosidase (substrate: 4-nitrophenyl-D-glucopyranoside) [45] and acid phosphatase (substrate: p-nitrophenylphosphate) [41], [46] were used as markers for endoplasmic reticulum and flagellar membranes, respectively. Protein content was measured by the Lowry assay (Bio-Rad).

### Antibodies and Western blotting

Proteins from aliquots of gradient fractions were precipitated with cold acetone and resuspended in denaturing SDS-PAGE buffer according to standard procedures. Separation of proteins by SDS-PAGE was performed under reducing conditions using 12.5%, w/v, acrylamide Criterion Precast gels containing 18 wells (Bio-Rad). For Western blotting, the proteins were transferred from gels to nitrocellulose membrane using Bio-Rad Trans-Blot Turbo equipment and Trans-Blot Turbo Transfer Packs (Midi Format, 0.2 µm nitrocellulose). The transfers were done using preprogrammed protocols. The membranes were blocked by 3%, w/v, bovine serum albumin in Tris-buffered saline (TBS) containing 0.2%, v/v, Tween 20 for at least 1 h at room temperature with gentle shaking, and incubated with the appropriate primary antibodies overnight at +4°C, followed by detection with horseradish peroxidase conjugated anti-rabbit or anti-mouse IgG (Santa Cruz Biotechnology). Polyclonal antisera, raised in rabbits, against aldolase (marker for glycosomes) and vacuolar pyrophosphatase (marker for acidocalcisomes; a gift by Dr. R. Docampo, University of Georgia, USA) were used as secondary antibodies. A monoclonal antibody against HSP60 (marker for the mitochondrial matrix) [47] was a gift by Dr. F. Bringaud (Université Bordeaux Segalen, France).

### Electron microscopy

Aliquots of the corresponding fractions of an Optiprep gradient were subjected to several rounds of concentration using a ‘Centriprep’ filter (cut-off 10 kDa) device (Millipore) and gradual dilution by homogenization buffer. This procedure was aimed to remove the gradient former (Iodixanol) and to concentrate particles while avoiding osmotic damage of glycosomes (see ref. [17] for details). The resulting samples were mixed with an equal volume of 2%, v/v, glutaraldehyde prepared in homogenization buffer. After fixation overnight at 4°C, the particles were sedimented at 20 000 *g* for 30 min, and the pellets were processed for transmission electron microscopy as described [16], [17].

### Detection of channel-forming activity

Channel-forming activities of glycosomal and mitochondrial preparations were registered using a Planar Lipid Bilayer Workstation equipped with a BP-535 amplifier and 8 pole low-pass Bessel filter (Warner Instruments). Acquisition and analysis were performed using pCLAMP software (Axon Instruments). The method is based on reconstitution of solubilized membrane proteins into an artificial lipid bilayer followed by detection of an electrochemical current arising due to insertion of proteins with channel-forming capability. Fractions collected from Optiprep or sucrose gradients were diluted 3–5 fold with 20 mM MOPS buffer, pH 7.2 (final concentration of protein: 0.01–0.05 mg/ml) and treated with detergents: 0.5%, w/v, Genapol X-080 or 0.2%, w/v, Fos-choline (final concentrations) by rotating for 2 h at 4°C. After centrifugation at 100 000 *g* for 40 min, the resulting supernatant was immediately used for registration of the channel-forming activity. Two experimental approaches were exploited for activity detection: multiple channel recording (MCR) and single channel analysis (SCA) [48]. MCR allows the registration of a large number of pore-forming events with detection of their conductance at a certain holding potential. This is a useful test for ‘pilot’ quantitative estimation of the presence of different channel-forming activities in membrane preparations. SCA was applied for detailed characterization of the properties of a certain channel type. The method allows an electrophysiological analysis of a single channel molecule. Measurements were performed in chambers with two compartments separated by a wall containing a tiny hole which was covered by an artificial membrane. The Ag/AgCl electrodes were connected to the compartments via 3 M KCl-agar bridges. The bath solutions contained 3 M KCl, 20 mM Tris-Cl, pH 7.8, and 2 mM DTT in both compartments (unless otherwise stated). The protein sample was added only into the *trans* compartment and the membrane currents were measured at a holding potential of +10 mV (unless otherwise stated). The other details of the measurements have been described [12], [13], [18] and some modifications are mentioned in the legends to Figures 3, 4, 5 and 6.

## Supporting Information

Figure S1 **SCA of a low-conductance channel.** (**A**) Current-voltage relationship averaged from SCA of five channels showing current rectification at negative voltages. The current amplitudes were measured after stepwise changes of the voltage (±20 mV). Bath solution (panels **A**, **B**, and **C**) contained 3 M KCl in both chambers. (**B**) The current-voltage relationship of the low-conductance channel in response to the shown voltage-ramp protocol (**B1**) or low-speed linear increase (**B2**, upper panel) and decrease (**B2**, lower panel) of the holding potential. All three protocols were applied to the same single channel. Note that the channel is open at all applied potentials. (**C**) Dependence of the current-voltage profile of low-conductance channels on the side of their insertion into an artificial membrane. Upper panel: A typical current-voltage relationship of the channel inserted from the *cis*-side of the membrane. Lower panel: Relative frequency of insertion of the low-conductance channels depending on the sample application to chamber compartments facing either the *trans* or *cis* side of an artificial membrane. The current-voltage relationship of each inserted channel was verified using the voltage-ramp protocol (see Figure S1 **C**, upper panel). The relative number of channels displaying current rectification at negative (filled bars) or positive (gray bars) holding potentials is shown. The total number of insertion events registered was 56 and 42 for *trans* and *cis* compartments, respectively.
(TIF)

Figure S2 **Glycosomal super-large-conductance channels.** (**A**) Current trace showing the insertion of two low-conductance channels (marked by asterisks) followed by the appearance of a stable super-large-conductance channel with current amplitude over 300 pA (3.0 M KCl, +10 mV). The dashed line indicates a current level (zero) before insertion of the channels. (**B**) Insertion of a highly unstable super-large conductance channel (1.0 M NH_4_Cl, +10 mV). The lower trace represents a timescale-expanded current recording of the upper trace. Direct transition of the current amplitude from near maximal to zero (marked by asterisk) indicates insertion of a single channel or channel cluster rather several separate channels. (**C**) Current-voltage relationship of a single super-large-conductance channel in response to the indicated voltage-ramp protocol (1.0 M NH_4_Cl at both sides of the membrane). The current amplitude of the channel before applying the voltage-ramp protocol was 120 pA at +10 mV. The appearance of multiple current amplitude transitions indicates the clustered nature of the super-large-conductance channel.
(TIF)
