# Supplementary figures and images for: Channel-Forming Activities in the Glycosomal Fraction from the Bloodstream Form of Trypanosoma brucei
Source: PLoS One. 2012 Apr 10;7(4):e34530. doi: 10.1371/journal.pone.0034530 (PMC3323538; doi:10.1371/journal.pone.0034530)

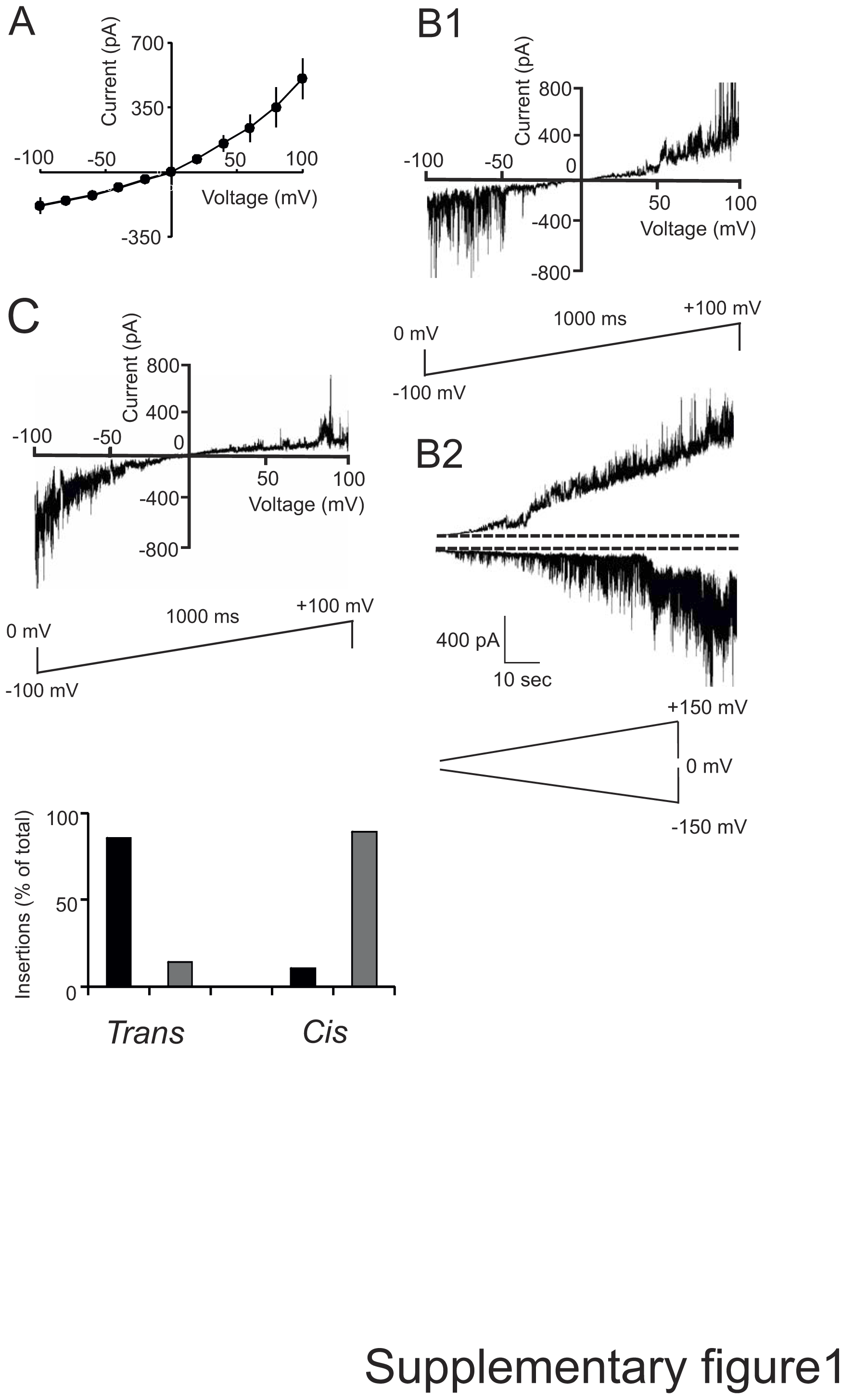

Supplement: Figure S1 — SCA of a low-conductance channel. (A) Current-voltage relationship averaged from SCA of five channels showing current rectification at negative voltages. The current amplitudes were measured after stepwise changes of the voltage (±20 mV). Bath solution (panels A, B, and C) contained 3 M KCl in both chambers. (B) The current-voltage relationship of the low-conductance channel in response to the shown voltage-ramp protocol (B1) or low-speed linear increase (B2, upper panel) and decrease (B2, lower panel) of the holding potential. All three protocols were applied to the same single channel. Note that the channel is open at all applied potentials. (C) Dependence of the current-voltage profile of low-conductance channels on the side of their insertion into an artificial membrane. Upper panel: A typical current-voltage relationship of the channel inserted from the cis-side of the membrane. Lower panel: Relative frequency of insertion of the low-conductance channels depending on the sample application to chamber compartments facing either the trans or cis side of an artificial membrane. The current-voltage relationship of each inserted channel was verified using the voltage-ramp protocol (see Figure S1 C, upper panel). The relative number of channels displaying current rectification at negative (filled bars) or positive (gray bars) holding potentials is shown. The total number of insertion events registered was 56 and 42 for trans and cis compartments, respectively. (TIF) [file pone.0034530.s001.tif]

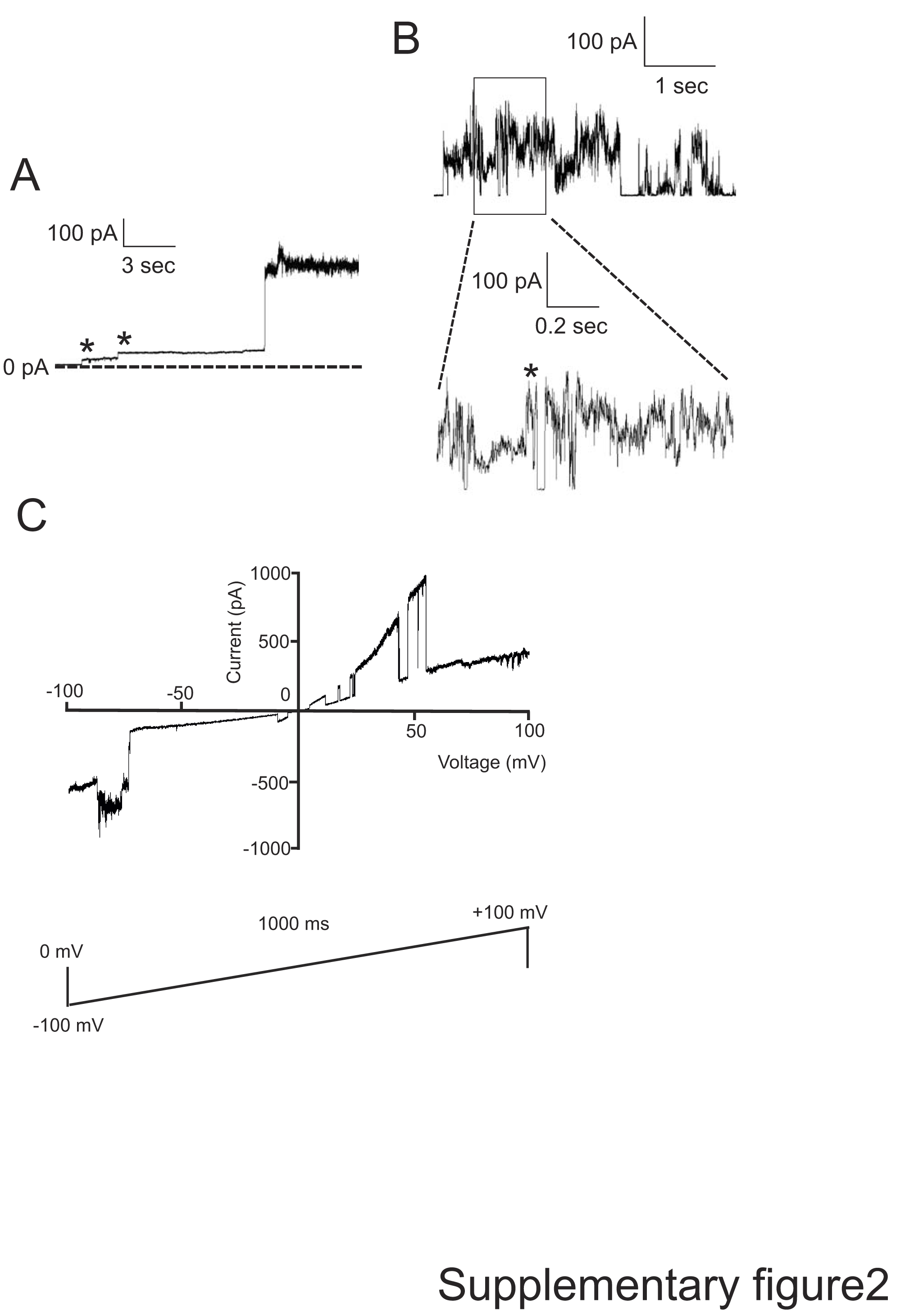

Supplement: Figure S2 — Glycosomal super-large-conductance channels. (A) Current trace showing the insertion of two low-conductance channels (marked by asterisks) followed by the appearance of a stable super-large-conductance channel with current amplitude over 300 pA (3.0 M KCl, +10 mV). The dashed line indicates a current level (zero) before insertion of the channels. (B) Insertion of a highly unstable super-large conductance channel (1.0 M NH4Cl, +10 mV). The lower trace represents a timescale-expanded current recording of the upper trace. Direct transition of the current amplitude from near maximal to zero (marked by asterisk) indicates insertion of a single channel or channel cluster rather several separate channels. (C) Current-voltage relationship of a single super-large-conductance channel in response to the indicated voltage-ramp protocol (1.0 M NH4Cl at both sides of the membrane). The current amplitude of the channel before applying the voltage-ramp protocol was 120 pA at +10 mV. The appearance of multiple current amplitude transitions indicates the clustered nature of the super-large-conductance channel. (TIF) [file pone.0034530.s002.tif]
